# Opinions and Suggestions on Nematode Faunal Analysis

**DOI:** 10.2478/jofnem-2024-0049

**Published:** 2024-12-24

**Authors:** Howard Ferris, Ingrid Varela Benavides

**Affiliations:** Department of Entomology and Nematology, University of California, Davis, California 95616, USA; Instituto Tecnológico de Costa Rica, San Carlos, Costa Rica

**Keywords:** Food web enrichment, structure, functions, services, succession, auto-regeneration, herbivory, faunal analysis

## Abstract

We briefly review the history and development of recognizing nematode assemblages as indicators of environmental conditions. We highlight the effects of spatio-temporal successional changes in nematode assemblages on the auto-regeneration of ecosystem functions after disturbance. We expand on the need for herbivory components in the analysis of soil nematode assemblages in recognition of the important impact of plant parasitism on the resources and productivity of the soil system. Finally, we point out some important areas of research that would enhance the process and value of nematode faunal analysis. We include an evaluation of the current potential for molecular assessment of nematode abundance and function and for the application of artificial intelligence in automated nematode identification.

## History and Foundational Principles

Humans, and perhaps their progenitors, have likely been aware of the indicator characteristics of nematodes since before recorded history, first as evidence of human and animal parasitism by the presence of nematodes in feces, skin lesions, and sputum and, with advances in microscopy, of free-living soil and freshwater forms. With the discovery and description of new species and habitats came the recognition that differences in feeding habits within the assemblage structure provide evidence of current or previous resource availability and the favorability of conditions for the resident taxa. Hence, whether stated explicitly, awareness of the indicator potential of nematode assemblages evolved.

Nematodes fulfill various trophic roles and perform important functions within the food webs that they inhabit. They are convenient indicators of similar functions other organisms perform in their food webs and are well-documented indicators of ecosystem conditions (Du Preez et al., 2022). Bastian (1865; 1866) opined that “free nematoids will be found to constitute one of the most widely diffused and abundant groups in the whole animal kingdom.” He commented on their abundance in a wide range of terrestrial, freshwater, and marine habitats. Cobb (1915), almost lyrically, declared that the composition and size of nematode assemblages indicate the utilization of the world’s resources by its inhabitants. So, the seeds of the indicator potential of nematodes were sown and germinated.

### Dimensions 1 and 2 of nematode faunal analysis: structure and enrichment

A huge conceptual advance in nematode indicator potential was the recognition by Bongers (1990) and associates (e.g., De Goede et al., 1993) that the nematode fauna can be categorized into five structural guilds based on the growth rates, survival characteristics, and stress tolerance of the taxa. The guild categorization recognized r-strategists with high productivity at one end of the colonizer-persister (c-p) series and K-strategists with lower productivity, greater longevity, and stress intolerance at the other end. The c-p series provided the basis for the Maturity Index (MI) as a synoptic model of ecosystem characteristics based on the taxon structure of the nematode assemblage. By the early 1990s, a cohesive foundation for faunal analysis was emerging (Bongers, 1990; Korthals et al., 1996; Bongers and Ferris, 1999) (Box 1).

Box 1. **Basic Principles of Nematode Faunal Analysis Models and their Validation**

1. **Enrichment Opportunists**
  - Nematode genera/species with very high consumption, metabolic, and production rates
  - Become dormant under adverse environmental and resource conditions
2. **Basal Fauna**
  - Slower activity, metabolism, and reproduction
  - Tolerant of adverse conditions
3. **Structure Indicators: Omnivores and Predators**
  - Larger bodies, slower growth and production rates, long life cycles
  - Intolerant of adverse conditions
4. **Characteristics of Structural Guilds**
  - Differ in metabolic resource partitioning
  - Differ in sensitivity to stress and disturbance at different rates
  - Recover from stress and undergo structural and functional succession at different rates

**Figure 1:**
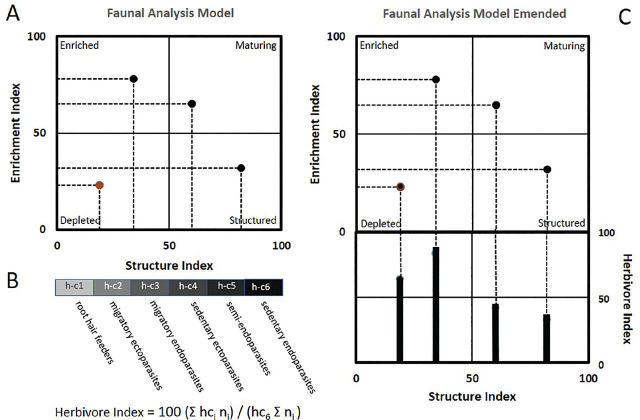
A: Nematode Faunal Analysis Model per Ferris et al., 2001) with sample data for four locations; B: Components of an Herbivore Index; C: Emended Faunal Analysis Model with Herbivore Index incorporated with sample data for four locations.

Ferris et al. (2001) separated the c-p series into three categories: a basal group, an enrichment group, and a structured group. We consider the First Dimension of Faunal Analysis to be the “K component,” the abundance of those nematodes that constitute the structure trajectory, characterized by their longevity and survival capabilities. The Second Dimension of Faunal Analysis is the enrichment trajectory, or “r component,” defined by the growth rate attributes of enrichment opportunistic taxa. The enrichment and structure indices are calculated separately, allowing the now familiar four-quadrat graphical model (Fig. 1A). Du Preez et al. (2022) provide background rationale and details for developing the MI and its derivative successor models.

### Dimension 3: carbon turnover and ecosystem services

The species consortia of the guilds of the nematode assemblage that comprise the basal, enrichment, and structure trajectories of the faunal analysis model differ in their contributions to ecosystem functions and services. The magnitude of those contributions is the Third Dimension of Faunal Analysis and can be calculated as the Metabolic Footprints of the component organisms (Ferris, 2010). The generally vermiform shapes of nematodes and the standardized morphometric characteristics used in their description facilitate the assessment of body volume and weight (Andrássy, 1956). Metabolic Footprints are inferred from prescribed coefficients, which, together with nematode morphometric parameters, allow estimations of nematode carbon metabolism and their production of body structure and offspring.

Taxonomic resolution at the family level in faunal analysis is problematic for biomass measurements. Although some families of nematodes have a smaller size range, 293 representatives of the family Dorylaimidae, as an example, have an average weight of 12.1 μg with a standard deviation of 28.4 and a coefficient of variation of 234% (Nemaplex.ucdavis.edu). The family mean mass is not a precise or reliable indicator of the weight of many genera and species. If the most abundant taxon of a family in a sample is of a size lower than the mean, biomass calculations based on average-sized individuals will be inflated; if greater, they will be deflated. That argues for taxonomic resolution and identification below the family level. A similar case has been made for differences in the functional attributes among genera within nematode families (Yeates, 1994). Integrative analysis of anatomical, morphometric, and molecular characteristics, perhaps aided by artificial intelligence developments, has enormous potential in identifying nematode taxa at the genus and species levels. That will allow reliable assignments to functional guilds and ease of application of faunal analysis. However, as always, speed and efficiency are of the essence.

Since buccal and pharyngeal structures of soil, freshwater, and marine nematodes provide a supportable basis for inference of feeding habits (Yeates et al., 1993), the third dimension recognizes the trophic habits within structural guilds of the c-p series. It builds on the functional implications for the soil ecosystem. As in other components of the faunal analysis model, assumptions and generalizations bridge the absence of empirical biological studies. The feeding habits of omnivore and predator nematodes are often inferred from combinations of oral structures and opportunistic observation. Nematodes that feed on other organisms have the potential, as predators, to regulate the abundance and activity of their prey (Steel and Ferris, 2016). However, quantitative relationships are uncertain as predator nematodes may be either ingesters or piercers, depending on their oral structures (Yeates et al., 1993). Some ingesters appear to be specifically adapted to feeding mainly on nematode prey. Still, unfiltered, open-mouthed feeding must involve the intake of organisms other than nematodes. Piercers, too, may be quite non-specific in their prey selection. Further experimentation and observation are warranted.

The criteria for assigning structure indicator taxa to c-p categories are somewhat vague (Bongers, 1990; Bongers & Bongers, 1998; Ferris et al., 2001). They include body size, inferred life course duration, and sensitivity to stress, all of which might be considered K-strategy characteristics. We suggest that a quantitative measure of relative K-strategy characteristics might be provided by the proportion of ingested carbon partitioned into reproduction in relation to that utilized for the growth and respiration of the nematode body. An approximation of that would be the volume or biomass of the adult female reproductive tract in relation to that of the whole body. Although authors sometimes provide details of ovary length, they do not indicate ovary volume from which mass might be estimated. From the sparse data available, it seems, however, that measures of the length of the ovary in relation to body size are around 25% for c-p5 species, somewhat larger for c-p4 species, and still larger for c-p3 species. Much more precise measures or relative volumes or mass would be necessary to validate and adopt such quantitative criteria or to include them along with more subjective characters.

#### An important subcategory, dimension 3a: the herbivory channel:

In formulating the MI, Bongers (1990) omitted plant-feeding taxa but calculated a separate Plant-Parasitic Index (PPI). He noted that when plants are good hosts for c-p3 plant parasites, the relationship between MI and PPI may be inverse (Bongers et al., 1997; Du Preez et al., 2022). On the other hand, in ecosystems with high plant diversity, the inverse relationship between MI and PPI may not occur (Neher and Campbell, 1996). Yeates (1994) and others argued for an alternative, ΣMI, in which plant-parasitic species are included.

The abundance and nature of plant-feeding nematodes impact the productivity of soil ecosystems and provide resources for predator organisms. The contribution to soil nutrient availability of root-hair feeders and other less-damaging parasites may offset any damage caused to plant growth. We believe an important enhancement of the faunal analysis model is the inclusion of a Herbivory Index (HI) based on the feeding habits and damage potential of plant-parasitic species. Interpretation of the herbivory component would be aided by developing a framework consistent with other dimensions of the faunal analysis model.

Yeates et al. (1993) recognized six h-c categories of plant-feeding nematodes based on their feeding habits. We use the h-c designation for terminological consistency with the c-p designations. We propose using the h-c categories to develop a weighting system based on the probable carbon turnover rates (Table 1A). The body weights of adult females are determined from morphometric data and are available as feeding category averages in Nemaplex (Nemaplex. ucdavis.edu). Refinements of the weighting system will occur as new information on feeding habits and damage potential of taxa emerges. We calculate the herbivory channel by weighing the abundance of nematodes in each category as a percentage of the carbon being processed if all nematodes present were in h-c6, the sedentary endoparasites. So, HI =100 (Σ hc_i_ n_i_) / (hc_6_ Σ n_i_), where i represents the 1 to 6 h-c weight classes of plant-feeding nematodes present in the assemblage (Table 1B). That allows a maximum value for carbon processed through the herbivory channel of 100. The Herbivory Index is integrated into the Emended Faunal Analysis Model using the Structure Index as the abscissa (Figs. 1 B,C). Some interpretive criteria are provided in Box 2; see also, Ferris et al., 2001.

**Table 1A.** Herbivore impact channel in Nematode Faunal Analysis with damage potential based on the average body mass of adult females in each h-c category (Fig. 1B) (Body mass data from Nemaplex accessed June 2024).

**h-c category**	**Female Body Mass μg hc_i_**	**Number of observations**
1. Root hair feeders	0.031	483
2. Migratory ectoparasites	0.008	274
3. Migratory endoparasites	0.581	350
4. Sedentary ectoparasites	0.656	1012
5. Semi-endoparasites	0.128	412
6. Sedentary endoparasites	34.942	606

**Table 1B.** Sample calculation of Herbivore Index, HI = (100 (Σ hc_i_ n_i_) / (hc_6_ Σ n_i_) for integration with Emended Nematode Faunal Analysis Model – Figs. 1 B,C)

**Genus**	**Functional Class**	**Sample Abundance n_i_**	**Weight hc_i_**	Weighted. Abundance hc_i_*n_i_
*Tylenchorhynchus*	hc_2_ Migratory ectoparasites	54	0.008	0.432
*Pratylenchus*	hc_3_ Migratory endoparasites	13	0.581	42.423
*Belonolaimus*	hc_4_ Sedentary ectoparasites	73	0.656	7.872
*Rotylenchulus*	hc_5_ Semi-endoparasites	12	0.128	1.664
*Meloidogyne*	hc_6_ Sedentary endoparasites	45	34.942	1572.39
Total Abundance (Σ n_i_)		197		
Total Weighted Abundance (Σ hc_i_ n_i_)				1624.77
Herbivore Index (100 (Σ hc_i_ n_i_) / (hc_6_ Σ n_i_))				23.60

Box 2: **Dimension 3A of Emended Nematode Faunal Analysis Model: inclusion of the herbivory channel**

**HI ≤ 50:** Herbivory Channel regulated by host status of plants to most damaging taxa, or regulated by predation or adverse environmental conditions

**HI > 50:** Herbivory Channel not regulated by host status; plants present are favorable hosts for the most damaging taxa, not regulated by predation or adverse environmental conditions

**Enrichment Quadrat:** Disturbed food webs; N-enriched; low C:N ratios; bacterial decomposition; low food web connectance

**Maturing Quadrat:** Low to moderately disturbed food webs; N-enriched; low C:N ratios; integrated bacterial and fungal decomposition; moderate food web connectance

**Structured Quadrat:** Undisturbed food webs; moderately enriched; moderate to high C:N ratios; fungal decomposition; high food web connectance

**Depleted Quadrat:** Food webs stressed; resources depleted; fungal decomposition; high C:N ratios; low food web connectance

Determination of the HI will require a few extra steps in the nematode abundance assessment. The abundance of ectoparasitic plant feeders can be determined through the extraction process used for free-living taxa. The abundance of sedentary and semi-endoparasitic nematodes can be determined as the number of egg masses, female bodies, or cysts on the root surface. If migratory endoparasites are present, mist chamber extraction of a root sample will also be necessary.

### Dimension 4 of nematode faunal analysis: spatio-temporal succession

Sometimes, the suggested interpretation of the Nematode Faunal Analysis models may seem counterintuitive or even erroneous. Although the concepts summarized in Box 1 seem logical and defensible based on our knowledge of nematode biology and ecology, reassessment of any model structure and parameter determination are always warranted. However, we believe that problems of interpretation occur when insufficient consideration is given to previous disturbances, to the variability of conditions and resources in the soil environment, and the dynamic nature of nematode populations and assemblages.

*Ecological Succession* is an important dimension to consider in applying and interpreting Nematode Faunal Analysis (Ettema and Bongers, 1993). The communities in which nematodes are interdependent are not static; they are constantly in rate-variable flux. Succession occurs in biological assemblages, and all component organisms are affected by, and respond to, changes in others. Further, abiotic and biotic environmental conditions regulate metabolic activity and affect interactions and succession among organisms (Poorter et al., 2024). Nutrient content and litter decomposition rates affect the decomposer community’s structure and the organisms that depend on it for resources (Schneider et al., 2012; Fabian et al., 2017; Canessa et al., 2021). Those changes are reflected in succession in the abundance and structure of the nematode assemblage (Ruess and Ferris, 2004).

The diversities of both nematode species and their functional attributes facilitate maximum exploitation of the resources available in different micro-patches of the soil (Ferris and Tuomisto, 2015). The diversity of guilds of a nematode assemblage (Interguild Diversity) expands the range of ecosystem services performed; species diversity within a functional guild (Intraguild Diversity) expands the contribution of species consortia to the magnitude of the service provided by that guild. Consequently, species diversity within and among functional guilds is a key element of the biological component of soil health. Understanding soil health and ecosystem function requires, besides knowledge of species diversity, assessment of the range of functions currently performed in the system and the abundance of organisms performing those functions. The diversity of the organisms and their environment, the rates at which resources are depleted, and the environmental factors that drive those rates determine the structural and functional succession of the nematode assemblage.

Besides succession at the field or landscape level, interguild and intraguild succession occur at the patch level. In fact, succession and its functional consequences at the landscape level are the integral of such factors across all patches in that landscape. Drivers of succession occur in all dimensional axes of the faunal analysis model. In the enrichment trajectory, following resource amendment, intraguild succession occurs when resources and environmental conditions are suitable for participant taxa, for example, the functional guild of opportunistic bacterivores. In that case, the relative abundances of taxa in the guild change over time in conjunction with changes in temperature and moisture conditions and the adaptation to those conditions of the taxa present. Taxa become predominant when temperature and moisture conditions are in synchrony with their ecophysiological attributes and population growth rates (Ferris et al., 1995; 1996a; 1997).

**Figure 2:**
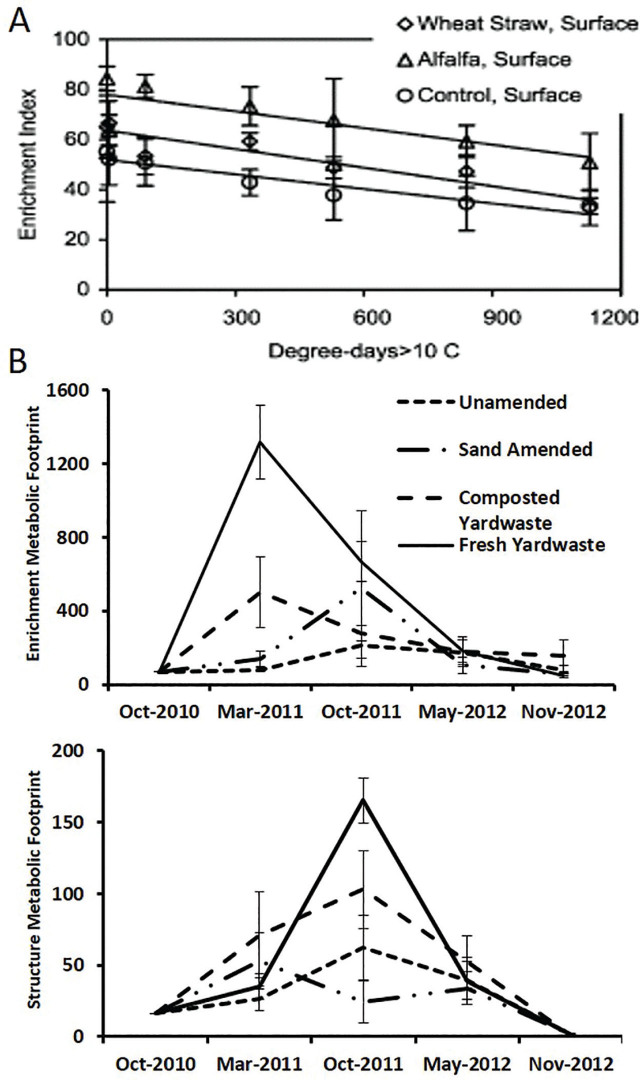
A: Effect on Enrichment Index of resource diminution over 9 months (data from Ferris and Matute, 2003); B: Succession in the Enrichment and Structure Footprint for 2 years following resource amendment. Based on a field trial by Dr. James Downer, Hansen Research and Extension Center, Ventura County, California.

As resource quality and availability diminish, interguild succession takes place. Taxa better adapted to current resource quality and availability predominate, e.g., c-p2 bacterivores and fungivores replace c-p1 taxa (Ferris et al., 1996b). With the diminution of resource quality, decomposition pathways change from bacterial to fungal, and fungivores increase proportionally; the Enrichment Index declines over time (Fig. 2A; Ferris and Matute, 2003). In the structure trajectory, intraguild succession occurs with a change in physical environmental conditions and probably with prey availability. The taxa in the c-p4 and c-p5 structural guilds increase at slower rates than those in the enrichment categories; they may have slower metabolic rates and lower partitioning of resources into the production of offspring (Figs. 2B). Also, following a disturbance, resource availability and distribution may filter through a stabilizing process before suitable resources are available in patches accessible and favorable for the disturbance-sensitive taxa. Functional interpretation of nematode faunal analysis requires consideration of the rate and direction of such changes. Usually, a single sampling period is insufficient, and a time course perspective is necessary.

#### Anthropogenic influences on Succession at the Landscape Level: Reversion and Regeneration

Reversion is a practice in which fields are retired from harvested production because their soils are considered damaged or depleted by conventional or high-input production practices. The area may be allowed to revert to the natural vegetation of that landscape with the hope that, in time, the physical, chemical, and structural nature of the soil will recover. Regeneration is a more managed conservation and rehabilitation process whereby resources are supplied that are considered necessary for the recovery of the soil. Practices include returning waste products of agricultural production to the soil and adding composted material from exogenous sources. Major goals include topsoil improvement, enhancing soil biodiversity, improving nutrient cycling, water porosity and drainage, biosequestration, and general enhancement of the productivity and viability of the soil. Degeneration, on the other hand, refers to changes in the soil that adversely affect those characteristics and generally reduce soil health by replacing its biological attributes with mineral fertilizers, pesticides, and tillage (Mitchell et al., 2024).

The productivity of anthropogenically modified or regenerative systems may require continued nurturing. Enhancements of the Structure and Enrichment Indices by organic amendment of field plots over a period of seven years were reversed in a single year during which organic amendments were terminated, and conventional agricultural practices were applied (Berkelmans et al., 2003). Unless the soil has an abundance of soil carbon, resources supplied by single applications of organic amendments are followed by a stimulus of the Enrichment Footprint and then a successional stimulus of the Structure Footprint, followed by a general decline in both as resources become depleted (Fig. 2B).

**Figure 3:**
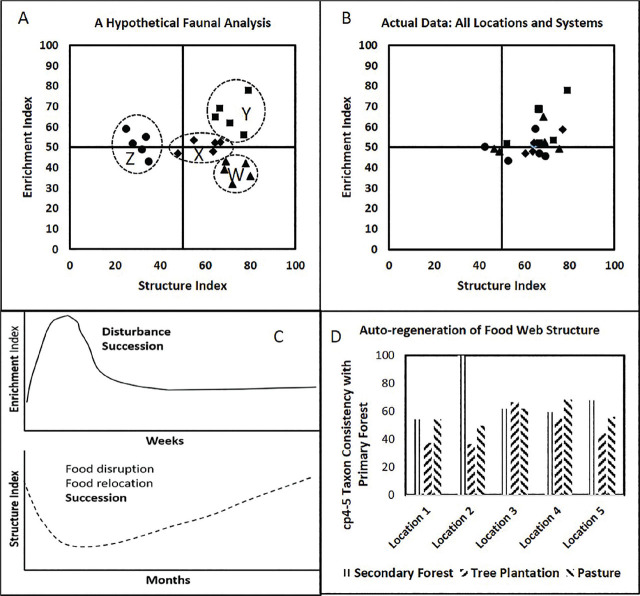
A: Hypothetical effects of primary forest clearing; letters indicate expected locations of the intersection of enrichment and structure trajectories after the disturbances, W: residual primary forest, X: secondary forest, Y: tree plantation, Z: pasture; B: Actual Faunal Analysis of residual primary forest, secondary forest, tree plantation and pasture.; C: Hypothetical auto-regeneration of enrichment and structure indicator components of the soil food web after a major disturbance, note time scale differences; D: Consistency of c-p 4 and 5 taxa 3 to 30 years after a disturbance as a percentage of those in primary forest. Data from Varela-Benavides et al. (2022).

Herein, we use the term auto-regeneration to describe the hypothesized natural sequence of recovery in soil ecosystems, and their functional attributes, where residual soil carbon stocks remain and are amplified by new sources of rhizodeposition. We believe that auto-regenerative processes are accelerated in the humid tropics. In a study of the effects of clearing primary tropical forests and allowing regrowth of secondary forests or converting them to either tree plantations or pasture, analysis of nematode assemblages in the new systems was conducted in five locations in a national park system in Costa Rica (Varela-Benavides et al., 2022). Faunal analyses were based on 100 composite samples of 10 cores each, taken in the same year at each location. The expectation, based on the faunal analysis model, was for an enhancement of the Enrichment Index as soil was disturbed and decomposition resources were made available to soil organisms, and a diminution of the Structure Index depending upon the amount of disturbance involved in conversion to the new soil ecosystem (Fig. 3C). The analyses of nematode assemblages in the systems at the five locations involved the identification and enumeration of nematodes of some 135 taxa, 55 of which were considered abundant or very abundant. Rather than clearly reflecting the hypothesized degree of disturbance (Fig. 3A), the analyses revealed that the Structure and Enrichment Index were quite similar across locations and systems (Fig. 3B).

An important variable not considered in the Costa Rican study was the successional events between the original disturbance and the time of sampling of the systems. In this case, the intervals at the various locations between clearing the primary forest and sampling for nematodes in the new ecosystems ranged from 3 to 30 years. We believe that is enough time for auto-regenerative processes to stabilize to a steady state of ecosystem functioning, particularly in a humid tropical environment (Fig. 3C).

Conversion of primary forests to other production systems involves harvesting timber, clear-cutting vegetation, uprooting stumps, and burning residual debris. Following that, there may be some land-leveling with heavy equipment and either secondary forest regrowth or the establishment of commercial tree crops or grasses for pasture. While all those are significant disturbances, none would be enough to eliminate resident nematode species except, perhaps, for extremely host-specific plant parasites. Rather, organic matter decomposition pathways would become re-established. However, in different micro-patches of the soil environment, enrichment-opportunistic nematode taxa would flourish in a relatively short timeframe (Fig. 3C). Consequently, resources would become available for survivors of cohorts of disturbance-intolerant nematode omnivores and predators in the c-p4 and c-p5 structural guilds. Over varying periods, depending on the ecophysiological and behavioral characteristics of organisms in the various structural guilds, auto-regeneration of the soil food web would occur, probably initially with variable and chaotic fluctuations of organism abundance but eventually stabilizing to a functional equilibrium of resource availability and utilization (Fig. 3C). If, however, removal of the primary forest and its residues involved the use of toxic materials to certain components of the soil food web, regeneration of higher and sensitive trophic levels might not occur, or would occur very slowly (e.g., Korthals et al., 1996; 1998).

The subsurface residues of the cleared forest provide widely dispersed masses of carbon sources that gradually become separated into patches of diminishing sizes through decomposition processes at different rates. The multipartite succession of organisms would occur in each patch at different rates, which is determined partly by the status of the residual carbon-based source and rhizodeposition from new plant sources. When considering the whole system subjected to disturbance and regeneration, the individual populations in each patch constitute a series of metapopulations (Hanski and Ovaskainen, 2000). Measurement of the integral biological structure of the complex distribution of patches is, of course, a challenge. Soil sampling devices encounter diverse groups of patches, resulting in variable and unreliable assessments of species presence and abundance. However, from repeated composites of such samples, successional trends in the nematode metapopulations emerge (Fig. 3C).

Supporting the auto-regeneration hypothesis is that, based on the composite sampling procedures in the Costa Rican study, even with their inherent variability, between 35 and 60% of the disturbance-sensitive c-p4 and c-p5 taxa of the primary forest soil in each location were detected in the disturbed systems (Fig. 3D). Further, the metabolic footprints of predator and omnivore taxa of the c-p4-5 stress-intolerant groups were related to resource prey availability indicated by the Enrichment plus Herbivory Footprints (analysis not shown). In other words, the auto-regenerative process was autonomous in the ecosystems that became established during the 3 to 30 years that elapsed after removing the primary forest.

As a caveat to the auto-regeneration hypothesis, we believe that recovery of the systems would not occur or would occur very slowly if suppressed by anthropogenic influences or by natural or unnatural disasters that result in prolonged conditions toxic to higher c-p group nematodes. Suppression of auto-regeneration can be inferred from many studies. It occurs when soils contaminated by residues from industrial activities or by residues and spills from mining operations remain toxic to sensitive structure-indicator species (Korthals et al., 1996; 1998; Sánchez-Moreno et al., 2006; Shao et al., 2008). As a general observation, the abundance of c-p4-5 taxa is usually very low in soils subjected to long-term conventional agriculture. In those soils, unlike the experience in the conversion of primary forest in Costa Rica, the frequency of occurrence of structure-indicator taxa is much lower than encountered in surrounding undisturbed soils (Sánchez-Moreno and Ferris, 2007; Sánchez-Moreno et al., 2008; 2009). Efforts to re-introduce such taxa from undisturbed areas are unsuccessful, at least in the short term, possibly due to changes in soil structure, a significant reduction in root biomass and soil organic carbon, or effects of persistent accumulation of mineral fertilizers (Tenuta and Ferris, 2004; DuPont et al., 2009; Glover et al., 2010).

## Future development of nematode faunal analysis

The MI and its various derivative forms have been applied in around 1,200 studies (Du Preez et al., 2022). The Faunal Analysis Model is just that, a Model. It represents our simplified concept of the complex realities of ecological systems. Like all such models, it is assembled with parameter values determined empirically or based on estimates and simplifying functions that we hope are logical. After testing and verification that the model, with various adaptations and extensions as outlined herein, functions as we expect, it will continue to be validated and interpreted by application in real-world systems. If it proves consistently unreliable, it may be modified or discarded; if reliable, it will be accepted and widely applied (see Du Preez et al., 2022).

There are some areas of research and study that are needed to improve the resolution of the faunal analysis system. In no specific order of importance, they include:

1. Refinement and verification of functional and trophic group assignment in taxa for which there is insufficient biological and ecological knowledge.
2. Determination of the effect of errors or differences of opinion in identifying a genus or higher taxon.
3. Further refinement of the third dimension of faunal analysis – the magnitude of effects based on abundances, body size, and metabolic activity of participant taxa on estimates of carbon utilization and throughput in the soil food web.
4. Some specific data gaps and verification currently needed:
  - Family, genus, species level functional and trophic group assignments for presumed plant-feeding nematodes of Tylenchidae, Aphelenchidae, Belondiridae, and Nordiidae.
  - Life course and productivity determinations for most nematode taxa, particularly the biology, importance, identification, and structural and functional guild assignments of the huge and diverse Dorylaimida.
  - Clarification of specific criteria for assigning taxa to c-p3, c-p4, and c-p5 structural guilds.
5. Further development of the third and fourth dimensions of the nematode faunal analysis profile:
  - Integration into the model of the abundance of plant-feeding nematodes, weighted by their body size and damage potential to plant productivity, into the faunal analysis.
  - Successional and regenerative rates and trajectories under different environmental conditions.
  - Integrating molecular and artificial intelligence tools for higher-resolution nematode identification and functional magnitude determinations for nematode assemblages.

Molecular applications in nematode faunal analysis are still in their infancy and will require further technological advances. Although 959 cells have been determined in the adult hermaphrodite *Caenorhabditis elegans* (Sulston and Horvitz, 1977; White et al., 1982), the number of cells in most nematode species is unknown. A seemingly reasonable assumption is that nematodes with larger body sizes have more cells than smaller nematodes and, consequently, more copies of species-specific gene sequences. That might lead to the simplistic hypothesis that if the number of species-specific sequences is divided by the body weight of a species as determined from morphometric calculations (Nemaplex.ucdavis.edu), the quotient should provide an approximation of the abundance of each species present in the sample. However, the issue of ploidy is a huge problem. For example, although *C. elegans* is a diploid organism, intestinal cells and syncytial cells of the hypodermis are polyploid, and 159 intestinal and hypodermal cells of an adult may contain 2100 genome copies (Hedgecock and White, 1985). In comparing *C. elegans* with eight other rhabditid species, Flemming et al. (2000) found that the ploidy of hypodermal nuclei varies significantly across species and life stages. The issues of genome copies and population stage structure present enormous problems in using metabarcoding for estimating individuals’ abundance or calculating ecophysiological metrics.

An optical recognition application of Artificial Intelligence shows some potential in faunal analysis. The process combines high-resolution imaging, multi-spectral sensing, large-bore flow cytometry, and machine learning to extract, isolate, count, and identify soil organisms in a high-throughput, high-resolution, non-destructive, and reproducible manner (Filgueiras et al., 2023). Of course, first, the machine must learn to identify nematodes. In that process, large numbers of individuals of a given nematode species are used in a “training set,” and images are recorded from all possible angles in 3-dimensional space. That requires large numbers of pure cultures of individual species, and the machine can only recognize those species that it has learned to identify. That is a challenge in application to species-rich environments, but perhaps morphotype identification would be an initial step. Again, much developmental work will be necessary in adaptation of AI flow cytometry techniques for nematode faunal analysis.

Changes in the assignment of taxa to families or c-p groups, or decisions on their feeding habits, create difficulties in comparison of faunal analyses across time or among studies and locations. Some assignment standardization is achieved through adherence to the data provided by online calculators, e.g., Sieriebriennikov et al. (2014). However, such tools may be saddled with their own built-in errors. When logic and empirical evidence suggest changes, a review of the basis for such changes may be necessary. We support the suggestion of a commission of ecologists of the International Federation of Nematology Societies as an arbiter of such changes, a form of established peer-review process. However, perhaps we inflate the importance of such considerations and step beyond the bounds of the utility of faunal analysis. Certainly, we should not disrupt the processes of consideration, hypothesis, verification, and validation. That would impose *rigor mortis* on this important area of research and application.

## References

[j_jofnem-2024-0049_ref_001] Andrássy I. (1956). Die rauminhalst and gewichtsbestimmung der fadenwurmer, (Nematoden). Acta Zoologica Academi Sciences. Hungary.

[j_jofnem-2024-0049_ref_002] Bastian H. C. (1865). Monograph on the Anguillulidae, or Free Nematoids, Marine, Land, and Freshwater; with Descriptions of 100 New Species. Transactions of the Linnean Society of London.

[j_jofnem-2024-0049_ref_003] Bastian H. C. (1866). On the anatomy and physiology of the nematoides, parasitic and free; with observations on their zoological position and affinities to echinoderms. Philosophical Transactions of the Royal Society of London MDCCCLXVI.

[j_jofnem-2024-0049_ref_004] Berkelmans R., Ferris H., Tenuta M., van Bruggen A. H. C. (2013). Effects of long-term crop management on nematode trophic levels other than plant feeders disappear after 1 year of disruptive soil management. Applied Soil Ecology.

[j_jofnem-2024-0049_ref_005] Bongers T. (1990). The maturity index: an ecological measure of environmental disturbance based on nematode species composition. Oecologia.

[j_jofnem-2024-0049_ref_006] Bongers T., Bongers M. (1998). Functional diversity of nematodes. Applied Soil Ecology.

[j_jofnem-2024-0049_ref_007] Bongers T., Ferris H. (1999). Nematode community structure as a bioindicator in environmental monitoring. Trends in Ecology & Evolution.

[j_jofnem-2024-0049_ref_008] Bongers T., van der Meulen H., Korthals G. (1997). Inverse relationship between the nematode maturity index and plant parasite index under enriched nutrient conditions. Applied Soil Ecology.

[j_jofnem-2024-0049_ref_009] Canessa R., van den Brink L., Saldaña A. (2021). Relative effects of climate and litter traits on decomposition change with time, climate and trait variability. Journal of Ecology.

[j_jofnem-2024-0049_ref_010] Cobb N. A. (1915). Nematodes and their relationships. USDA Yearbook of Agriculture 1914.

[j_jofnem-2024-0049_ref_011] De Goede R. G. M., Bongers T., Ettema C. H. (1993). Graphical presentation and interpretation of nematode community structure: c-p triangles. Mededelingen Faculteit Landbouwkundige en toegepaste biologische wetenschapen Univesiteit Gent.

[j_jofnem-2024-0049_ref_012] DuPont S. T., Ferris H., Van Horn M. (2009). Effects of cover crop quality and quantity on nematode-based soil food webs and nutrient cycling. Applied Soil Ecology.

[j_jofnem-2024-0049_ref_013] Du Preez G., Daneel M., De Goede R. (2022). Nematode-based indices in soil ecology: Application, utility, and future directions. Soil Biology and Biochemistry.

[j_jofnem-2024-0049_ref_014] Ettema C. H., Bongers T. (1993). Characterization of nematode colonization and succession in disturbed soil using the Maturity Index. Biology and Fertility of Soils.

[j_jofnem-2024-0049_ref_015] Fabian J., Zlatanovic S., Mutz M., Premke K. (2017). Fungal–bacterial dynamics and their contribution to terrigenous carbon turnover in relation to organic matter quality. ISME Journal.

[j_jofnem-2024-0049_ref_016] Ferris H. (2010). Form and function: Metabolic footprints of nematodes in the soil food web. European Journal of Soil Biology.

[j_jofnem-2024-0049_ref_017] Ferris H., Bongers T., de Goede R. G. M. (2001). A framework for soil food web diagnostics: extension of the nematode faunal analysis concept. Applied Soil Ecology.

[j_jofnem-2024-0049_ref_018] Ferris H., Eyre M., Venette R. C., Lau S. S. (1996a). Population energetics of bacterial-feeding nematodes: Stage-specific development and fecundity rates. Soil Biology and Biochemistry.

[j_jofnem-2024-0049_ref_019] Ferris H., Lau S., Venette R. (1995). Population energetics of bacterial-feeding nematodes: Respiration and metabolic rates based on CO_2_ production. Soil Biology and Biochemistry.

[j_jofnem-2024-0049_ref_020] Ferris H., Matute M. M. (2003). Structural and functional succession in the nematode fauna of a soil food web. Applied Soil Ecology.

[j_jofnem-2024-0049_ref_021] Ferris H., Tuomisto H. (2015). Unearthing the role of biological diversity in soil health. Soil Biology and Biochemistry.

[j_jofnem-2024-0049_ref_022] Ferris H., Venette R. C., Lau S. S. (1996b). Dynamics of nematode communities in tomatoes grown in conventional and organic farming systems, and their impact on soil fertility. Applied Soil Ecology.

[j_jofnem-2024-0049_ref_023] Ferris H., Venette R. C., Lau S. S. (1997). Population energetics of bacterial-feeding nematodes: Carbon and nitrogen budgets. Soil Biology and Biochemistry.

[j_jofnem-2024-0049_ref_024] Filgueiras C. C., Kim Y., Wickings K. G., El Borai F., Duncan L. W., Willett D. S. (2023). The Smart Soil Organism Detector: An instrument and machine learning pipeline for soil species identification. Biosensors and Bioelectronics.

[j_jofnem-2024-0049_ref_025] Flemming A. J., Shen Z. Z., Cunha A., Emmons S. W., Leroi A. M. (2000). Somatic polyploidization and cellular proliferation drive body size evolution in nematodes. Proceedings of the National Academy of Sciences of the United States of America.

[j_jofnem-2024-0049_ref_026] Glover J. D., Culman S. W., DuPont S.T. (2010). Harvested perennial grasslands provide ecological benchmarks for agricultural sustainability. Agriculture, Ecosystems & Environment.

[j_jofnem-2024-0049_ref_027] Hanski I., Ovaskainen O. (2000). The metapopulation capacity of a fragmented landscape. Nature.

[j_jofnem-2024-0049_ref_028] Hedgecock E. M., White J. G. (1985). Polyploid tissues in the nematode *Caenorhabditis elegans*. Developmental Biology.

[j_jofnem-2024-0049_ref_029] Korthals G., Popovici J., van Megen H. H. N. (1998). Soil nematodes in heathland around a zinc s melter near Budel, The Netherlands. Nematode Communities of Northern Temperate Grassland Ecosystems.

[j_jofnem-2024-0049_ref_030] Korthals G. W., Ende A., Megen H., Lexmond T. M., Kammenga J. E., Bongers T. (1996). Short-term effects of cadmium, copper, nickel and zinc on soil nematodes from different feeding and life-history strategy groups. Applied Soil Ecology.

[j_jofnem-2024-0049_ref_031] Mitchell J. P., Cappellazzi S. B., Schmidt R. (2024). No-tillage, surface residue retention, and cover crops improved San Joaquin Valley soil health in the long term. California Agriculture.

[j_jofnem-2024-0049_ref_032] Neher D. A., Campbell C. L. (1996). Sampling for regional monitoring of nematode communities in agricultural soils. Journal of Nematology.

[j_jofnem-2024-0049_ref_033] Poorter L., van der Sande M. T., Amissah L. (2024). A comprehensive framework for vegetation succession. Ecosphere.

[j_jofnem-2024-0049_ref_034] Ruess L., Ferris H. (2004). Decomposition pathways and successional changes. Nematology Monographs and Perspectives.

[j_jofnem-2024-0049_ref_035] Sánchez-Moreno S., Camargo J. A., Navas A. (2007). Ecotoxicological assessment of the impact of residual heavy metals on soil nematodes in the Guadiamar River Basin (Southern Spain). Environmental Monitoring and Assessment.

[j_jofnem-2024-0049_ref_036] Sánchez-Moreno S., Ferris H. (2007). Suppressive service of the soil food web: Effects of environmental management. Agriculture, Ecosystems & Environment.

[j_jofnem-2024-0049_ref_037] Sánchez-Moreno S., Nicola N. L., Ferris H., Zalom F. G. (2009). Effects of agricultural management on nematode–mite assemblages: Soil food web indices as predictors of mite community composition. Applied Soil Ecology.

[j_jofnem-2024-0049_ref_038] Sánchez-Moreno S., Smukler S., Ferris H., O’Geen A. T., Jackson L. E. (2008). Nematode diversity, food web condition, and chemical and physical properties in different soil habitats of an organic farm. Biology and Fertility of Soils.

[j_jofnem-2024-0049_ref_039] Schneider T., Keiblinger K. M., Schmid E. (2012). Who is who in litter decomposition? Metaproteomics reveals major microbial players and their biogeochemical functions. ISME Journal.

[j_jofnem-2024-0049_ref_040] Shao Y., Zhang W., Shen J. (2008). Nematodes as indicators of soil recovery in tailings of a lead/zinc mine. Soil Biology and Biochemistry.

[j_jofnem-2024-0049_ref_041] Sieriebriennikov B., Ferris H., de Goede R. G. M. (2014). NINJA: An automated calculation system for nematode-based biological monitoring. European Journal of Soil Biology.

[j_jofnem-2024-0049_ref_042] Steel H., Ferris H. (2016). Soil nematode assemblages indicate the potential for biological regulation of pest species. Acta Oecologica.

[j_jofnem-2024-0049_ref_043] Sulston J. E., Horvitz H. R. (1977). Post-embryonic cell lineages of the nematode, Caenorhabditis elegans. Developmental Biology.

[j_jofnem-2024-0049_ref_044] Tenuta M., Ferris H. (2004). Sensitivity of nematode life-history groups to ions and osmotic tensions of nitrogenous solutions. Journal of Nematology.

[j_jofnem-2024-0049_ref_045] Varela-Benavides I., Abolafia J., Guevara-Mora M., Peña-Santiago R., Ferris H. (2022). Nematode assemblages in four ecosystems of Parque Nacional del Agua, Costa Rica. Applied Soil Ecology.

[j_jofnem-2024-0049_ref_046] White J. G., Horvitz H. R., Sulston J. E. (1982). Neuron differentiation in cell lineage mutants of *Caenorhabditis elegans*. Nature.

[j_jofnem-2024-0049_ref_047] Yeates G. W. (1994). Modification and qualification of the nematode maturity index. Pedobiologia (Jena).

[j_jofnem-2024-0049_ref_048] Yeates G. W., Bongers T., De Goede R. G. M., Freckman D. W., Georgieva S. S. (1993). Feeding habits in soil nematode families and genera-an outline for soil ecologists. Journal of Nematology.

